# Complete Genome Sequence of a Human Cytomegalovirus Strain AD169 Bacterial Artificial Chromosome Clone

**DOI:** 10.1128/genomeA.00091-16

**Published:** 2016-03-31

**Authors:** Eleonore Ostermann, Michael Spohn, Daniela Indenbirken, Wolfram Brune

**Affiliations:** Heinrich Pette Institute, Leibniz Institute for Experimental Virology, Hamburg, Germany

## Abstract

The complete sequence of the human cytomegalovirus strain AD169 (variant ATCC) cloned as a bacterial artificial chromosome (AD169-BAC, also known as HB15 or pHB15) was determined. The viral genome has a length of 230,290 bp and shows 52 nucleotide differences compared to a previously sequenced AD169*var*ATCC clone.

## GENOME ANNOUNCEMENT

The cloning of herpesvirus genomes as a bacterial artificial chromosome (BAC) allows the conservation of a genetically defined clone and its genetic modifications using bacterial recombination systems (1–3). The first human cytomegalovirus (HCMV, human herpesvirus 5) strain cloned as a BAC was the widely used laboratory strain AD169 (ATCC VR-538). The BAC clone pHB5 contains the genome of the AD169 strain with a deletion in the US2 to US6 region, which was introduced for stable insertion of the BAC cassette (4). The deletion was later repaired by reinsertion of the deleted sequence and insertion of loxP sites adjacent to the BAC cassette to allow for Cre-mediated excision of the BAC cassette (5). The BAC clone containing the complete AD169*var*ATCC sequence was named AD169-BAC and was used for the construction of numerous HCMV mutants in many laboratories around the world. In some publications, the AD169-BAC is referred to as HB15 or pHB15 (6–10).

In the present study, AD169-BAC DNA was isolated from the *Escherichia coli* strain DH10B, purified using a Nucleobond Xtra-Midi column (Macherey-Nagel) following the manufacturer’s instructions, and resuspended in 10 mM Tris-HCl, pH 8.0. 400 ng of BAC DNA was used for library preparation using a NEBNext Ultra DNA sample preparation kit (NEB) according to the manufacturer’s recommendations. Diluted libraries were paired-end sequenced (2 × 250 cycles) with an Illumina MiSeq sequencer generating 1.8 million paired reads. Reads were trimmed with Trimmomatic (11), using a minimum length of 40 bp (MINLEN) and a quality-cutoff at the 3′-end of Q15 (TRAILING). The guided *de novo* assembly was done with SPAdes (12), using Human herpesvirus 5 AD169*var*UK (GenBank accession no. BK000394) as a guide (option untrusted contig). All other options were defaults.

The 238,831-bp AD169-BAC genome contained the sequence of the 8,037-bp BAC cloning vector inserted in the US2 open reading frame. The BAC cassette consists of an F plasmid replicon, a guanosine phosphoribosyl transferase (gpt) gene for selection in mammalian cells, and a chloramphenicol acetyltransferase (cat) gene for selection in *E. coli*, and an origin of transfer (oriT) (5). Compared to the AD169 sequences already published (GenBank Accession no. FJ527563, X17403, AC146999, and BK000394), this AD169-BAC is most similar to the AD169*var*ATCC clone pAD/Cre (13, 14) (GenBank accession no. AC146999). The AD169-BAC sequence differs in only 52 nucleotides from the pAD/Cre sequence, of which 36 are in intergenic or intronic regions, 2 are predicted to be silent, and 14 single nucleotide differences are predicted to affect the amino acid sequences of the viral proteins pUL32/pp150, pUL44, pUL92, pUL99, pIRL10, pIRL6, pIRL1, and pIRS1/pTRS1.

### Nucleotide sequence accession number.

This whole-genome sequence of strain AD169-BAC (HB15) has been deposited at GenBank under the accession no. KU317610.
